# The relationship between HbA_1c_ and ultrasound plaque textures in atherosclerotic patients

**DOI:** 10.1186/s12933-016-0422-5

**Published:** 2016-07-19

**Authors:** Xiao-Wei Huang, Yan-Ling Zhang, Long Meng, Ming Qian, Wei Zhou, Rong-Qin Zheng, Hai-Rong Zheng, Li–Li Niu

**Affiliations:** Paul C. Lauterbur Research Center for Biomedical Imaging, Institute of Biomedical and Health Engineering, Shenzhen Institutes of Advanced Technology, Chinese Academy of Sciences, Shenzhen, China; Department of Ultrasound, Third Affiliated Hospital, Sun Yat-sen University, Guangzhou, China

**Keywords:** Ultrasound imaging, Carotid plaque, Texture, HbA_1c_, Cardiovascular disease

## Abstract

**Objective:**

Diabetes mellitus (DM) is associated to the morphological and componential characteristics of atheromatous plaques. It has proven that plaque textures are related to plaque components and beneficial for atherosclerotic risk stratification. The aim of this study is to compare plaque textures in patients with and without DM, and examine the relationship between HbA_1c_ levels and the ultrasound plaque textures in atherosclerotic patients.

**Methods:**

A total of 136 participants (among them 66 are diabetic and 70 are non-diabetic) suffering from carotid plaques were included. About 300 texture features were extracted from the ultrasound images of plaques using the algorithms of histogram, absolute gradient, run-length matrix, gray-level co-occurrence matrix, autoregressive model and wavelet transform, respectively. Thirty optimal features were selected by the Fisher coefficient and the mutual information measure. The most discriminating feature (MDF) was obtained from the linear discriminant analysis for the optimal features. Linear regression model was performed to investigate the relationship between HbA_1c_ and MDF. The receiver operating characteristics (ROC) curve was further developed to validate the relation between the estimated HbA_1c_ (models output) and diabetes status.

**Results:**

A total of 12 texture features showed statistical difference between patients with and without DM. The MDF was significant higher in non-diabetic patients (0.326 ± 0.049) than diabetic patients (−0.346 ± 0.052) (p < 0.001). The optimal regression model (r = 0.348, p < 0.001) for HbA_1c_ included a constant (p < 0.001) and the MDF (p < 0.001). The areas under ROC curve used to estimate HbA_1c_ was 0.828.

**Conclusions:**

The results indicate that there is a quantitative relationship between the HbA_1c_ levels and plaque textures in ultrasonic images of atherosclerotic patients, which may suggest that texture analysis of the ultrasonic image of plaque is a promising method for evaluating the cardiovascular risk caused by DM in patients with plaques.

## Background

Diabetes mellitus (DM) enable to accelerate atherosclerosis and increase the risk of cardiovascular diseases [1, 2]. Carotid intima-media thickening (IMT) measured by ultrasound imaging and carotid artery elasticity evaluated by ultrasound radio frequency technology have been utilized for assessing the impact of DM on the plaques [3, 4]. These schemes, however, ignore some essential information about the plaque surface and morphology. Substantial evidences have indicated that quantitative textural analysis of the medical images can provide more useful diagnostic information [5], and is useful for atherosclerotic risk stratification [6]. Thus, it is significant to characterize carotid plaques using textures in patients with and without DM, which may help to evaluate the risk of cardiovascular events caused by DM.

DM are associated with the morphological and the componential characteristics of carotid plaques [3, 7]. In previous studies, Wilhjelm et al. [8] found that texture features of the ultrasound plaques was correlated with the histologically determined relative volume of soft materials. Niu et al. [9] indicated that texture features extracted from ultrasound images of the carotid arterial wall were useful in identifying arterial surface roughness. Rakebrandt et al. [10] reported that there are five texture classes match with the plaque contents including fibrin, elastin, calcium, haemorrhage and lipid. The above studies may imply that some plaque textures are different in patients with and without DM. Furthermore, HbA_1c_ is a risk factor for cardiovascular diseases in type 2 diabetes [11], and is related to the mortality in heart failure patients with diabetes [12]. However, few studies have investigated the relationship between HbA_1c_ and plaque textures.

The aim of this study is to compare plaque textures in patients with and without DM, and examine the relationship between HbA_1c_ levels and plaque textures in atherosclerotic patients.

## Subjects and methods

### Subjects

A total of 136 subjects (among them 66 are diabetic and 70 are non-diabetic) suffering from carotid plaques were investigated in present study. From October 2011 to February 2015, the patients received carotid artery examination at the department of ultrasound, the third affiliated hospital of Sun Yat-sen University. All participants provided the written informed consent. The study protocol was approved by the Institutional Review Board of the third affiliated hospital of Sun Yat-sen University (Guangzhou, China).

In this study, the diagnostic criteria for diabetes are defined as fasting plasma glucose (FPG) level of ≥7.0 mmol/L, and/or 2-h plasma glucose value of ≥11.1 mmol/L, and/or HbA_1c_ level of ≥6.5 %, and/or treatment with either hypoglycemic agents or insulin [13, 14]. Patients with acute or chronic infectious disease, alcohol or drug abuse, retinopathy, or uncontrolled hypertension were excluded. Information regarding age, gender, total number of plaques, systolic blood pressure (SBP), diastolic blood pressure (DBP), body mass index (BMI), total cholesterol, triglyceride, low density lipoprotein cholesterol, high density lipoprotein cholesterol, lipoprotein, apolipoprotein A1, apolipoprotein B100, FPG, HbA_1c_ and medication use were collected.

### Carotid ultrasonography

The study was performed by a specialized physician with 5 years vascular ultrasound working experience using a Toshiba AplioXG SSA-790A ultrasound Platform equipped with a 5–12 MHz linear-array transducer (PLT-805AT) and Esaote MyLab90 ultrasound Platform equipped with a 4–13 MHz linear-array transducer (LA523). The carotid artery was examined with the head tilted slightly upward in the mid-line position. The transducer was manipulated so that the near and far walls were parallel to the transducer footprint, and the lumen diameter was maximized in the longitudinal plane.

To improve the comparability of the plaque images obtained by different ultrasound systems at different settings, all images were standardized according to the scheme proposed by Sabetai et al. [15] before texture analysis. Furthermore, it is more clinically significant to focus on echolucent plaques, since these plaques are more potentially unstable than echo-rich plaques [16]. Gray-scale median (GSM) analysis is an objective and reproducible method for evaluating the echogenicity of carotid plaque [15]. In case there were multiple plaques in one individual, the plaques with the lowest GSM value among them was selected as the representative for the following texture analysis [17]. Two operators performed the GSM measurement independently, and the interoperator reproducibility was evaluated with a *kappa* value. The disagreement of the two operators were discussed and re-evaluated, then an agreement was finally achieved.

Many studies have shown that carotid intima-media thickening (IMT) is a high risk factor of the future cardiovascular events [18–20]. Maximum IMT (Plaque-IMTmax) was defined as the greatest axial thickness among the plaques in the carotid arteries [21, 22], and was measured in this study.

### Texture analysis

**Fig. 1:**
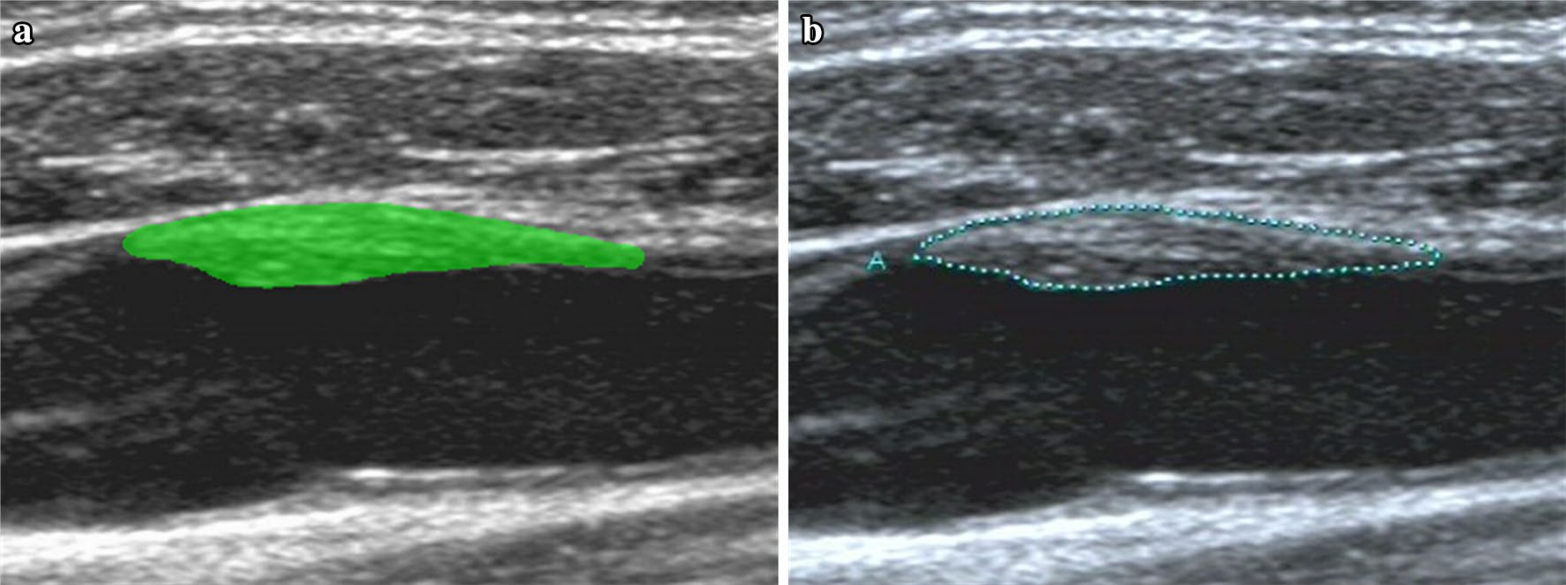
The region of interest selection. **a** The region of interest selection according to (**b**) using MaZda software. **b** Plaque defined by a specialized physician with 5 years vascular ultrasound working experience

MaZda is an effective tool for texture analysis and offers an approach for texture feature extraction, selection and reduction [23]. In MaZda, we can draw regions of interest with arbitrary shapes, as shown in Fig. 1. It provides six various algorithms, such as histogram, absolute gradient, run-length matrix, co-occurrence matrix, autoregressive model and wavelet for features extraction [5, 23]. In present study, about 300 texture features of carotid plaques were extracted using MaZda, as shown in Table 1.

**Table 1:** Texture features

*Histogram* (1) mean, (2) variance, (3) skewness, (4) kurtosis and (5) percentiles 1, 10, 50, 90 and 99 %
*Absolute gradient* (1) mean, (2) variance, (3) skewness, (4) kurtosis and (5) percentage of pixels with nonzero gradient
*Run-length matrix* (1) run-length nonuniformity, (2) grey-level nonuniformity, (3) long-run emphasis, (4) short run emphasis and (5) fraction of image in runs. Parameters computed for horizontal, 45° vertical and 135° orientation
*Co-occurrence matrix* (1) angular second moment, (2) contrast, (3) correlation, (4)sum of squares, (5) inverse difference moment, (6) sum average, (7) sum variance, (8) sum entropy, (9) entropy, (10) difference variance and (11) difference entropy. Parameters are computed for 4 orientations: (a, 0), (0, a), (a, a), (a, −a) and 5 distances: a = 1, 2, 3, 4, 5; between image pixels
*Autoregressive model* (1) model parameter vector includes 4 parameters and (2) standard deviation of the driving noise
*Wavelet* (1) Energy of wavelet coefficients in low-frequency subbands, (2) horizontal high-frequency subbands, (3) vertical high-frequency subbands and (4) diagonal high-frequency subbands at successive scales

### Texture feature selection and reduction

In order to select optimal features among the large number of texture features of the plaques from diabetic and non-diabetic patients, the methods based on Fisher coefficient and mutual information measure were used to select 15 optimal features, respectively. Furthermore, linear discriminant analysis (LDA) was implemented for the combined feature set, and the most discriminating features (MDF) were obtained.

#### Fisher coefficient

Fisher coefficient is defined as a ratio of between-class scatter *D* to within-class variance *V* [24]:

1 $$F = \frac{D}{V}{ = }\frac{{\frac{1}{{1 - \sum\nolimits_{k = 1}^{K} {P_{k}^{2} } }}\sum\nolimits_{k = 1}^{K} {\sum\nolimits_{j = 1}^{K} {P_{k} P_{j} (\mu_{k} - \mu_{j} )^{2} } } }}{{\sum\nolimits_{k = 1}^{K} {P_{k} v_{k} } }}$$

where $u_{i}$, $v_{i}$ and $P_{i}$ denote the mean, the variance and the priori probability of class *i*, respectively. Texture features with larger Fisher coefficient are selected as optimal features.

#### Mutual information measure

Mutual information (MI), a measure of dependence between two random variables, is defined as [25]:

2 $$MI\left( {X,Y} \right) = H\left( X \right) + H\left( Y \right) - H\left( {X,Y} \right)$$

where *X* and *Y* are random variables, *H* is the entropy. In case of *X* stores values of texture features and *Y* stores the classification decision. Then, a large MI between *X* and *Y* means that *X* is a useful texture features for classification. Then the MI for each texture features $f_{i}$ is calculated by [25, 26]:

3 $$MI(f_{i} ,d) = \sum\limits_{d = 1}^{{N_{b} }} {\sum\limits_{k = 1}^{Nc} {P\left( {f_{i}^{d} ,c_{k} } \right)\log_{2} \left[ {\frac{{P\left( {f_{i}^{d} ,c_{k} } \right)}}{{P\left( {f_{i}^{d} } \right)P\left( {c_{k} } \right)}}} \right]} }$$

where $d = c_{1} ,c_{2} , \ldots ,c_{{N_{C} }}$ means the class category, $N_{c}$ is the total number of class, $N_{b}$ is the number of histogram bins used for feature discretization, $f_{i}^{d}$ denotes discretized value of $f_{i}$.

#### Linear discriminant analysis

LDA is a useful method for feature reduction [24]. The aim of LDA is to find a transform matrix W such that the ratio of determinants $\frac{{\left| {W^{T} S_{B} W} \right|}}{{\left| {W^{T} S_{W} W} \right|}}$ is maximized. Where $S_{B}$ and $S_{W}$ are the between-class scatter matrix and the within-class scatter matrix. These matrices can be given as formulas (4, 5).

4 $$S_{B} = \frac{1}{M}\sum\limits_{k = 1}^{{N_{c} }} {M_{k} \left( {x_{i}^{(k)} - u^{(k)} } \right)\left( {x_{i}^{(k)} - u^{(k)} } \right)^{T} }$$

5 $$S_{W} = \frac{1}{M}\sum\limits_{k = 1}^{{N_{c} }} {\sum\limits_{i = 1}^{{M_{k} }} {(x_{i}^{(k)} - u^{(k)} )(x_{i}^{(k)} - u^{(k)} )^{T} } }$$

where $${\text{X}}_{i}^{(k)}$$ denotes the *i*-th pattern in class *k*$$(i = 1,2, \ldots ,M_{k} ),$$$$k = 1,2, \ldots ,N_{c}$$, $$u^{(k)}$$ is the mean vector of class *k.* It has proved that such a transform matrix $$\varPhi$$ is composed of eigenvectors corresponding to largest eigenvalues of $$S_{W}^{ - 1} S_{B}$$. The MDF can be obtained when the original data is transformed by the means of matrix $$\varPhi$$ as formula (6).

6 $$MDF_{i} = \varPhi^{T} (x_{i} - u)$$

### Statistical analysis

All statistical analysis was performed with PASW Statistics 18 and *p* less than 0.05 was considered statistically significant. All values were presented as the mean value ±SD, or real number of patients with the percentage in parentheses. Independent sample *ttest* was used to examine the baseline clinical parameters between the diabetic and non-diabetic patients. Pearson correlation analysis was conducted to investigate the relationship between HbA_1c_ and the variables including age, BMI, total number of plaques, SBP, DBP, plaque-IMTmax and MDF. Linear regression analysis was carried out by considering the HbA_1c_ as a dependent variable and regarding the MDF as independent variable. The optimized regression model was obtained to estimate the HbA_1c_. Further, the receiver operating characteristics (ROC) curve was developed to test the relationship between the estimated HbA_1c_ (models output) and diabetes status.

## Results

### Baseline characteristics of study participants

Table 2 describes the baseline characteristics of the study population (n = 136), which includes 66 patients with DM (age, mean ± SD, 67.8 ± 10.0 years) and 70 patients without DM (age, mean ± SD, 69.4 ± 9.8 years). The BMI (23.9 ± 3.1 vs 22.0 ± 2.8 kg/m^2^, p = 0.001), triglyceride (1.90 ± 1.57 vs 1.31 ± 0.89, *p* = 0.009 mmol/L), HbA_1c_ (8.87 ± 2.25 vs 5.50 ± 0.41 %, *p* < 0.001) and FPG (10.76 ± 4.26 vs 5.14 ± 0.70 mmol/L, *p* < 0.001) were significantly different in patients with and without DM. In the DM group, the ratio of patients using oral hypoglycemic agent, insulin, both oral hypoglycemic agent and insulin, and no drug for treatment were 50 % (n = 33), 21.2 % (n = 14), 9.1 % (n = 6), and 19.7 % (n = 13).

**Table 2:** Baseline characteristics of 136 subjects

Characteristics	Diabetes (n = 66)	Non-diabetes (n = 70)	*t*	*p*
Age, mean (SD)	67.8 ± 10.0	69.4 ± 9.8	−0.972	0.333
Male gender, n (%)	43 (65)	46 (66)	0.068	0.946
Total number of plaques	2.1 ± 1.2	2.0 ± 1.1	0.533	0.104
Hypertension, n (%)	43 (65)	44 (63)	0.277	0.783
CHD, n (%)	18 (27.3)	22 (31.4)	−0.528	0.598
SBP (mm Hg)	136.9 ± 19.0	139.1 ± 22.0	−0.642	0.522
DBP (mm Hg)	75.8 ± 9.2	76.7 ± 12.8	0.465	0.643
BMI (kg/m^2^)	23.9 ± 3.1	22.0 ± 2.8	3.437	0.001
Total cholesterol (mmol/L)	4.78 ± 1.20	4.46 ± 1.17	−1.548	0.116
Triglyceride (mmol/L)	1.90 ± 1.57	1.31 ± 0.89	2.677	0.009
HDL-cholesterol (mmol/L)	1.05 ± 0.28	1.13 ± 0.34	−1.484	0.140
LDL-cholesterol (mmol/L)	3.04 ± 0.97	2.86 ± 1.06	1.023	0.308
Lipoprotein A (mg/L)	211.9 ± 219.2	284.9 ± 288.7	−1.652	0.101
Apolipoprotein A1(g/L)	1.29 ± 0.31	1.31 ± 0.26	−0.362	0.718
Apolipoprotein B100 (g/L)	1.20 ± 0.47	1.17 ± 1.23	0.163	0.871
HbA_1c_ (%)	8.87 ± 2.25	5.50 ± 0.41	11.940	<0.001
FPG (mmol/L)	10.76 ± 4.26	5.14 ± 0.70	10.611	<0.001
Plaque-IMTmax (mm)	2.4 ± 0.8	2.5 ± 0.8	−0.635	0.527

Data are presented as the mean value ±SD or percentage of subjects

*CHD* coronary heart disease, *SBP* systolic blood pressure, *DBP* diastolic blood pressure, *BMI* body mass index, *HDL-cholesterol* high density lipoprotein cholesterol, *LDL-cholesterol* low density lipoprotein cholesterol, *FPG* fasting plasma glucose

### Plaques selection

There was a good agreement between the two operators in selecting the plaque with the lowest GSM value in one individual with multiple plaques. The interoperator reproducibility was 97.79 % (*kappa* value = 0.965), and the 3 controversial plaques were discussed and re-evaluated, then eventually an agreement was achieved.

### Texture selection and reduction

A total 30 optimal features were selected based on the Fisher coefficient and the mutual information measure. Among the optimal features, 12 features (7 feature extracted by run-length matrix and 5 features extracted by wavelet) were considered to be statistically different in patients with and without DM, as shown in Table 3. In order to reduce the number of the optimal features, LDA was further performed to gain the MDF. The MDF (−0.035 ± 0.052 vs 0.033 ± 0.050, *p* < 0.001) showed significant difference in the diabetic and the non-diabetic patients.

**Table 3:** Statistically different features between diabetic and non-diabetic plaques

Features	Non-diabetes (n = 70)	Diabetes (n = 66)	Fisher	MI	*t*	*p*
WavEnLH_s-4	756.21 ± 418.35	522.48 ± 385.45	0.342	0.109	3.383	0.001
Vertl_Fraction	0.883 ± 0.056	0.847 ± 0.096	0.213	0.036	2.630	0.010
135dr_ShrtREmp	0.921 ± 0.037	0.893 ± 0.0716	0.25	0.066	2.866	0.005
Vertl_ShrtREmp	0.915 ± 0.040	0.886 ± 0.072	0.243	0.046	2.810	0.006
WavEnLL_s-2	16,327 ± 4554	13,092 ± 7191	0.229	0.055	2.2733	0.007
WavEnLL_s-1	16,327 ± 4559	13,474 ± 7337	0.224	0.041	2.705	0.008
WavEnLH_s-3	465.39 ± 275.86	343.30 ± 247.33	0.220	0.039	2.712	0.008
135dr_Fraction	0.892 ± 0.052	0.858 ± 0.091	0.213	0.040	2.627	0.010
45dgr_ShrtREmp	0.918 ± 0.038	0.892 ± 0.073	0.210	0.038	2.606	0.011
WavEnLL_s-3	15,479 ± 4811	12,802 ± 7299	0.193	0.069	2.510	0.014
Horzl_ShrtREmp	0.816 ± 0.095	0.757 ± 0.169	0.191	0.048	2.491	0.014
45dgr_Fraction	0.887 ± 0.053	0.854 ± 0.095	0.184	0.040	2.444	0.016
MDF	0.033 ± 0.050	−0.035 ± 0.052	–	–	7.712	<0.001

WavEnLH_s-3 and WavEnLH_s-4 are energy of wavelet coefficients in vertical high-frequency subbands at scale 3 and 4; WavEnLL_s-1, WavEnLL_s-2 and WavEnLH_s-3 are energy of wavelet coefficients in low-frequency subbands at scale 1, 2 and 3; 45dgr_Fraction, Vertl_Fraction and 135dr_Fraction are fraction of image in runs computed for 45°, vertical and 135° orientation; Vertl_ShrtREmp, Horzl_ShrtREmp, 45dgr_ShrtREmp, Vertl_ShrtREmp and 135dr_ShrtREmp are short run emphasis computed for horizontal, 45° vertical and 135° orientation

*MDF* most discriminating feature, *MI* mutual information

### Pearson correlation of the study variables with HbA_1c_

Pearson correlation analysis was implemented to examine the relationship between HbA_1c_ and the variables including age, BMI, total number of plaques, SBP, DBP and MDF. Table 4 indicates that the HbA_1c_ is positively correlated with BMI (r = 0.182, *p* = 0.034), whereas HbA_1c_ is negatively correlated with the MDF (r = −0.348, *p* < 0.001).

**Table 4:** Pearson correlation of the study variables with HbA_1c_

		Age (year)	BMI (kg/m^2^)	TNP	SBP (mmHg)	DPB (mmHg)	Plaque-IMTmax (mm)	MDF
HbA_1c_ (%)	Pearson	−0.123	0.182	−0.022	−0.071	0.084	−0.097	−0.348
	*p*	^0.155^	^0.034^	^0.795^	^0.408^	^0.333^	0.262	^<0.001^

*BMI* body mass index, *TNP* total number of plaques, *SBP* systolic blood pressure, *DBP* diastolic blood pressure, *MDF* most discriminating feature

### Linear regression model for HbA_1c_

Table 5 shows that the optimal model for HbA_1c_ (r = 0.348, *p* < 0.001) is achieved by stepwise regression method. The model included a constant (*p* < 0.001) and the MDF (*p* < 0.001). The optimized regression equation can be described as formula (7) to estimate the HbA_1c_.

**Table 5:** Linear regression model of HbA_1c_ with the coefficients of the variables involved

Model	Variables/constant	Coefficients	*P*	95.0 % confidence interval	
				Lower bound	Upper bound
HbA_1c_ (r = 0.392, *p* < 0.001)	Constant	7.136	<0.001	6.766	7.506
	MDF	−13.297	<0.001	−19.407	−7.186

*MDF* most discriminating feature

7 $$Estimated\,{\text{HbA}}_{1c} = - 13.297*x_{1} + 7.136$$

where $x_{1}$ is the MDF.

**Fig. 2:**
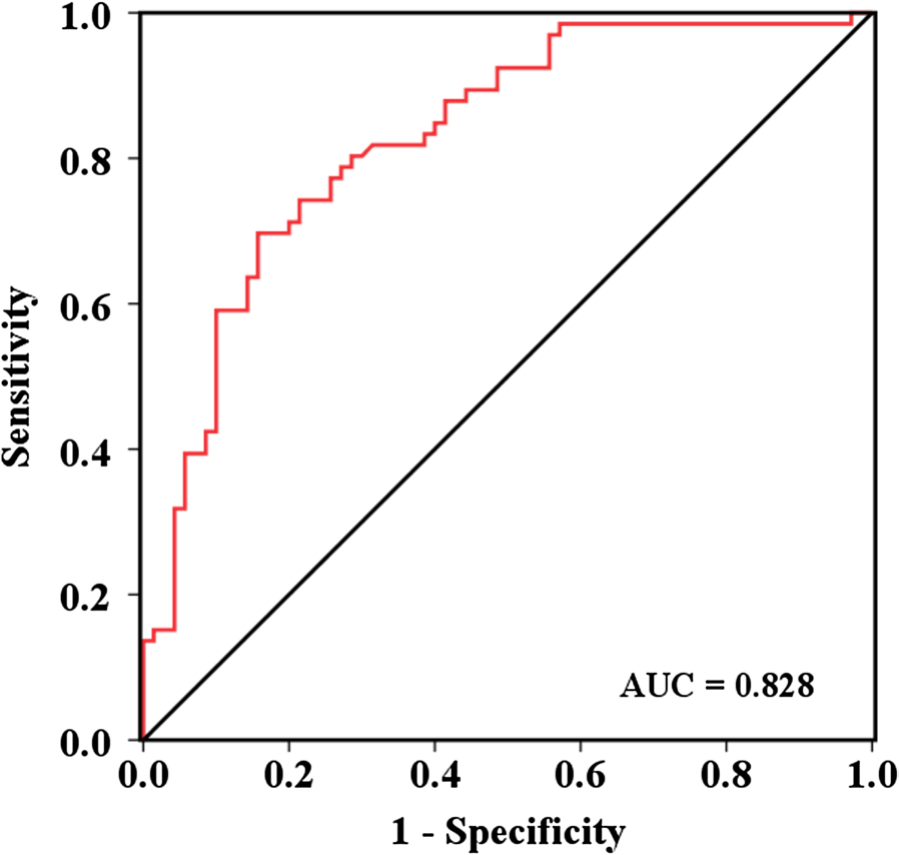
The receiver operating characteristics curve for validating the relationship between estimated HbA_1c_ and diabetes status. AUC represents areas under receiver operating characteristics curve

Further, the ROC curve was developed to validate the relationship between the estimated HbA_1c_ and diabetes status, and the area under the ROC curve was 0.828 (Fig. 2).

## Discussion

In this study, a total of 12 texture features showed significant difference in diabetic and non-diabetic patients. Previous studies indicated that MD impacted the vascular structure and function [27, 28], increased the IMT of common carotid artery [3], and modified the relationships between carotid plaque calcium, composition and inflammation [7]. The textural differences may be a candidate parameter reflecting the morphological and componential differences of the carotid plaques in patients with and without DM. The texture features including the fraction of image in runs computed for 45°, vertical and 135° orientation, and the short run emphasis computed for horizontal, 45°, vertical and 135° orientation were statistically higher in non-diabetic patients than diabetic patients. The fraction of image in runs is a measurement of the percentage of image pixels that are part of any of the runs considered for the matrix computing and it should have a low value for images with linear structure [5, 29]. Moreover, the short run emphasis, a feature emphasizing the short runs, is a measure of the proportion of short runs occurring in the image [5, 29]. Given the above, it may suggest that diabetes tend to impact plaques with a linear structure in the surface.

In previous studies, Virmani et al. [30] indicated that the BMI was significantly higher in diabetic patients (30.5 ± 7.41 kg/m^2^) than in non-diabetic patients (26.6 ± 5.4 kg/m^2^) (*p* = 0.001). Nozue et al. [31] showed that BMI was significantly different in the participants with MD or not (25.3 ± 3.9 vs 23.8 ± 2.7 kg/m^2^, *p* = 0.01). Marso et al. [32] indicated that the median triglycerides in acute coronary syndrome patients with MD was 144 mg/dL, whereas that in patients without MD was 101 mg/dL (*p* < 0.001). In the study of Lee et al. [33] reported that the median triglycerides in subjects with HbA1c ranged from 3.6 to 5.2, from 5.3 to 5.4, from 5.6 to 5.7, and from 5.8 to 6.4 were 109.1, 116.1, 120.2, and 127.9 mg/dL, respectively (*p* < 0.001). These findings are consistent with our results, in which the BMI (23.9 ± 3.1 vs 22.0 ± 2.8 kg/m^2^, *p* = 0.001) and triglycerides (1.90 ± 1.57 vs 1.31 ± 0.89 mmol/L, *p* = 0.009) are statistically different in the patients with and without DM.

Ultrasound IMT measurements were considered as a strong predictor of future cardiovascular events [18–20]. Recent studies suggested that the increased IMT was not an independent predictor of plaque development [34, 35]. Compare with carotid IMT, carotid plaque predicts cardiovascular events more accurately [36, 37]. In this study, the carotid plaque-IMTmax showed no statistical difference in patients with and without DM (2.4 ± 0.8 vs 2.5 ± 0.8 mm, *p* = 0.527), whereas the MDF was significant higher in diabetic patients (0.033 ± 0.050) than in non-diabetic patients (−0.035 ± 0.052) (*p* < 0.001). It may suggest that the MDF is more effective than carotid plaque-IMTmax in illustrating the difference of plaques in patients with and without DM. Additionally, MDF make it possible to evaluate the stability of the plaque. Compared with the conventional features (i.e. plaque-IMTmax) that are evaluated visually, the MDF is an abstract feature that extracted from texture features without visual evaluation.

HbA_1c_ testing reflects the average plasma glucose over the previous 2–3 months [38]. Mukai et al. [39] indicated that the crude average of the maximum carotid intima-media thickness increased significantly with rising quartiles of HbA_1c_. Daida et al. [40] examined the association between HbA_1c_ and plaque regression, and suggested that plaque regression was less pronounced in patients with high HbA_1c_ levels compare with those with low levels. In present study, we found a relationship between the HbA_1c_ levels and plaque textures in atherosclerotic patients, which may be useful for evaluating the impact of long-term blood glucose level on the carotid plaques. Furthermore, Eeg-Olofsson et al. [11] indicated that higher HbA_1c_ levels increased the risks of cardiovascular disease, coronary heart disease and total mortality. Ikeda et al. [41] showed that HbA_1c_ was an independent risk factor for cardiovascular disease. The relationship between the MDFs and HbA_1c_ may suggest that the MDFs can be used to assess cardiovascular risk, which may elevate the value of B-mode ultrasonography examination.

The main limitation was that this method could be applied only to patients with carotid plaques. The atheromatous plaque, however, was a common complication of diabetes and had a high prevalence in population [42, 43]. Besides, the number of subjects was relatively small in this study. A longitudinal, prospective study utilizing carotid ultrasound evaluation in a large number of diabetic patients with plaques is required to assess the precise prognostic value of plaque textures in determining future cardiovascular events caused by DM.

## Conclusion

In conclusion, the results of present study indicated that the textures of the carotid plaques were statistically different in patients with and without DM. The relationship between the HbA_1c_ levels and plaque textures may suggest that texture analysis of the ultrasound images of carotid plaques is a promising method to evaluate the risk of cardiovascular events caused by DM in patients with plaques.

## Abbreviations

DM: diabetes mellitus
MDF: most discriminating feature
ROC: receiver operating characteristics
IMT: intima-media thickening
FPG: fasting plasma glucose
SBP: systolic blood pressure
DBP: diastolic blood pressure
BMI: body mass index
GSM: gray-scale median
Plaque-IMTmax: maximum intima-media thickening
LDA: linear discriminant analysis
MI: mutual information
CHD: coronary heart disease
HDL-cholesterol: high density lipoprotein cholesterol
LDL-cholesterol: low density lipoprotein cholesterol
WavEnLH_s-3 and WavEnLH_s-4: energy of wavelet coefficients in vertical high-frequency subbands at scale 3 and 4
WavEnLL_s-1, WavEnLL_s-2 and WavEnLH_s-3: energy of wavelet coefficients in low-frequency subbands at scale 1, 2 and 3
45dgr_Fraction, Vertl_Fraction and 135dr_Fraction: fraction of image in runs computed for 45°, vertical and 135° orientation
Vertl_ShrtREmp, Horzl_ShrtREmp, 45dgr_ShrtREmp, Vertl_ShrtREmp and 135dr_ShrtREmp: short run emphasis computed for horizontal, 45°, vertical and 135° orientation
TNP: total number of plaques
